# Synthetic evolution of *Saccharomyces cerevisiae* for biomanufacturing: Approaches and applications

**DOI:** 10.1002/mlf2.12167

**Published:** 2025-02-23

**Authors:** Zhen Wang, Xianni Qi, Xinru Ren, Yuping Lin, Fanli Zeng, Qinhong Wang

**Affiliations:** ^1^ College of Science & Technology Hebei Agricultural University Cangzhou China; ^2^ Key Laboratory of Engineering Biology for Low‐carbon Manufacturing, Tianjin Institute of Industrial Biotechnology Chinese Academy of Sciences Tianjin China; ^3^ National Center of Technology Innovation for Synthetic Biology Tianjin China; ^4^ College of Life Sciences Hebei Agricultural University Baoding China

**Keywords:** biomanufacturing, metabolic performance, *Saccharomyces cerevisiae*, stress tolerance, synthetic evolution

## Abstract

The yeast *Saccharomyces cerevisiae* is a well‐studied unicellular eukaryote with a significant role in the biomanufacturing of natural products, biofuels, and bulk and value‐added chemicals, as well as the principal model eukaryotic organism utilized for fundamental research. Robust tools for building and optimizing yeast chassis cells were made possible by the quick development of synthetic biology, especially in engineering evolution. In this review, we focused on methods and tools from synthetic biology that are used to design and engineer *S. cerevisiae*'s evolution. A detailed discussion was held regarding transcriptional regulation, template‐dependent and template‐free approaches. Furthermore, the applications of evolved *S. cerevisiae* were comprehensively summarized. These included improving environmental stress tolerance and raising cell metabolic performance in the production of biofuels and bulk and value‐added chemicals. Finally, the future considerations were briefly discussed.

## INTRODUCTION

The world is on the cusp of an industrial revolution fueled by biotechnology and biomanufacturing. It is anticipated that synthetic biology will pave the path for a sustainable manufacturing industry. Based on synthetic biology, biomanufacturing has become the advanced mode for producing commercially important biomolecules for use in the agricultural, food, material, energy, and pharmaceutical industries. Biomanufacturing is greatly contributing to developing the low‐carbon economy and effectively reducing the excessive dependence on fossil resources, and is expected to completely reshape the development model of carbon‐based civilization, and trigger industrial evolution. Microorganisms are the core tools of biomanufacturing, and they are the focus of biomanufacturing R&D and industrial competition. *Saccharomyces cerevisiae*, the most well‐known and frequently employed microorganism, has a wide range of uses in the biochemical, biofuel, and fermented food industries^1^, ^2^. It is also a valuable model organism used extensively for research in genetics and molecular biology^3^. In recent decades, engineered *S. cerevisiae* has been comprehensively applied to convert renewable and cheap carbon sources into top value‐added chemicals and natural products^4^, ^5^. For industrial applications, *S. cerevisiae* often needs to achieve the highest substrate utilization and product formation and survive various stress conditions to achieve maximum economic benefits^6^. Hence, developing robust and stress‐tolerant *S. cerevisiae* to meet complex industrial fermentation conditions has drawn much more attention recently.

As the first fully sequenced eukaryotic organism, *S. cerevisiae* has undergone extensive molecular and cell biology research. The development of a *S. cerevisiae* cell factory to generate the desired products seems to be simple due to its multiple benefits, including genetic stability, clear genetic history, and the availability of a wide range of genetic tools^7^. Metabolic engineering provides a powerful strategy to reshape metabolic pathways and generate excellent microbial cell factories by introducing novel biosynthetic pathways and removing the competing pathways^8^. Although the ability of homologous recombination of *S. cerevisiae* is superior to most microbial cells, such as *Escherichia coli* and *Pichia pastoris*, genetic modification was still inefficient and laborious in *S. cerevisiae*. In recent years, the advances in genome editing technologies, especially the Clustered Regularly Interspaced Short Palindromic Repeats (CRISPR)/Cas system, have greatly enhanced our capacity to make targeted genome changes and boosted the design and engineering of *S. cerevisiae* ^9^. Despite the fact that metabolic engineering is frequently required to produce novel cell factories, introducing new pathways and rewiring native metabolism can amass intermediates and hazardous byproducts that put cells under stress conditions^6^. Furthermore, the circumstances utilized for production processes put the cells under additional stress conditions from factors like low pH, extreme temperature, or high osmosis^10^. The lack of total understanding of stress response mechanisms makes it difficult to improve complex traits of *S. cerevisiae* using metabolic engineering strategies. Therefore, it is crucial to develop novel evolution tools and strategies for acquiring the desired phenotype.

Microbial populations may adapt to long‐term exposure to severe settings by gaining important driver mutations that modify specific biological processes^11^. Evolutionary engineering has been developed as a useful strategy for improving strain phenotypes or physiological features, including environmental tolerance and product titers^12^. One of the most well‐developed and widely used evolutionary engineering is adaptive laboratory evolution (ALE), which consists of continuous cultivations over numerous generations with steady or increasing selective pressure^13^. With the advent of high‐throughput screening technologies and low‐cost whole‐genome sequencing, evolved mutants or populations can be quickly identified and analyzed to establish the genetic basis related to tolerance characteristics. As a result, various types of stress tolerance in *S. cerevisiae* have been addressed using ALE^14^. Although artificial selection‐driven spontaneous mutation may be sufficient to generate enough mutants for selective pressure to act upon, ALE is hard to cope with more complex traits and the “trade‐off” between “increased fitness of selective pressure” and “lost other traits”^6^.

**Figure 1:**
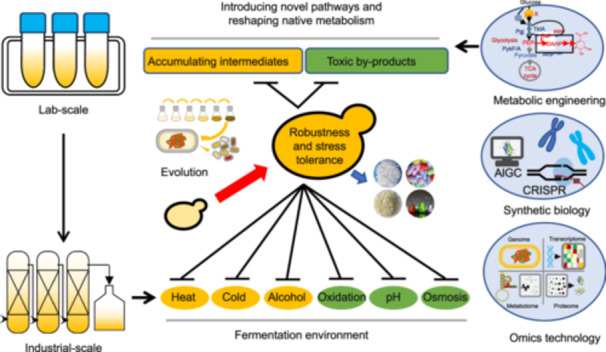
The development of *Saccharomyces cerevisiae* synthetic evolution. *S. cerevisiae* is a popular laboratory‐mode eukaryote, and adaptive laboratory evolution has been utilized to improve strain phenotypes, including robustness and stress tolerance. Cells must endure difficult fermentation settings, such as excessive temperatures, alcohol, and oxygen stress to maximize economic rewards. Metabolic engineering, synthetic biology, and omics technology enable the introduction of additional pathways and the altering of major metabolic pathways to construct great microbial cell factories.

The advances in synthetic biology technologies have considerably expanded the ability to design and develop cells with desired phenotypes in a short time^15^. In the past decade, synthetic biology has shown promise in understanding the evolution of gene expression and in the directed evolution of protein biocatalysts^16^, ^17^. Synthetic biological design also drew inspiration from observations of evolution. Advances in the synthetic biology field accelerate the evolution cycle and facilitate the engineering of increasingly complex traits in biological systems^18^. Hence, synthetic evolution is described as the iterative process of applying current synthetic biology techniques to diversify and select one or more specific genetic loci with the desired function or phenotype. Synthetic evolution is complementary to natural selection since it may be used to modify organisms in non‐natural contexts^19^ (Figure 1).

## SYNTHETIC EVOLUTION TOOLS AND STRATEGIES

Natural populations can adapt to a wide range of situations due to the chromosomal diversity seen among organisms, including *S. cerevisiae*. However, diverse genomic alterations and novel phenotypes are hard to develop in the laboratory on practical timescales. Traditional evolution methods, such as random mutagenesis with the treatment of mutagens, are commonly laborious, time‐consuming, and relatively inefficient. Synthetic evolution is becoming a more popular method for diverse genomic alternations and, therefore, overcoming industrial yeast's physiological limitations and boosting its robustness (Table 1). The technologies and strategies of synthetic evolution can mainly be summarized into three characteristics depending on whether DNA damage is caused and the exogenous template is employed in yeast *S. cerevisiae* cells: (1) template‐dependent synthetic evolution; (2) template‐free synthetic evolution; (3) transcriptional regulation‐based synthetic evolution.

**Table 1:** Methods and strategies of synthetic evolution for phenotypic disturbance in *Saccharomyces cerevisiae*.

Method and strategy	Principle of action	Advantage and disadvantage	Key application in *S. cerevisiae*	Ref.
Artificial transcription factors (artificial TFs)	Different specific zinc finger structures are expressed in fusion with activator/repressor domains to stochastically regulate the transcriptional levels of genes	**Advantage**: randomized regulation; targeting the whole genome; high generalizability between species; convenient plasmid library construction; and simple operation **Disadvantage**: zinc finger structure mismatch, resulting in a cumbersome targeting process; and difficult to construct and screen cloned phenotypically equivalent strains through reverse engineering	Thermotolerance, osmotolerance, and drug resistance	^20^
Global transcription machinery engineering (gTME)	Changes in the translation level of multiple proteins are achieved by introducing a library of transcription factor mutations	**Advantage**: targeting full transcript level changes; applicable across multiple species **Disadvantage**: huge number of targeted proteins; difficult to reverse engineer	Ethanol tolerance	^21^
CRISPR interference and CRISPR activation (CRISPRi/a)	Up‐regulating or down‐regulating the expression level of target genes using dead Cas9 (dCas9) or dCas9 fusion repressor/activator	**Advantage**: targeting specific loci for regulation **Disadvantage**: difficult to achieve genome‐wide regulation	Production of glycerol and 3‐dehydromangostinic acid	^22^, ^23^, ^24^
In vivo continuous evolution (ICE)	Reverse transcriptional transposons are error‐prone during reverse transcription and mutations are generated in the target sequence	**Advantage**: continuous evolution in vivo; simple operation **Disadvantage**: the transposition process increases the copy number of the target sequence, which affects the positive mutation screening; poor generalization between species; low efficiency	Evolution of butanol tolerance and xylose utilization	^25^
synthetic chromosome rearrangement and modification by LoxP‐mediated evolution (SCRaMbLE)	Genome rearrangement using loxP repeat sequences catalyzed by Cre recombinase	**Advantage**: large genomic segment modification **Disadvantage**: only be used in specific strains with pre‐incorporated loxP sequences	Carotenoid production, deoxypurple bacillin, xylose utilization, acetic acid, ethanol, and thermotolerance	^26^, ^27^, ^28^, ^29^, ^30^, ^31^, ^32^
A highly error‐prone orthogonal DNAP‐DNA plasmid pair (OrthoRep)	Specific mutation of cytoplasmic plasmid sequences using cytoplasmic plasmid‐specific DNA polymerase mutants	**Advantage**: target gene is completely orthogonal to the genomic mutation **Disadvantage**: tendency to favor mutations that favor growth	Anti‐malarial drug resistance studies	^33^

### Template‐dependent synthetic evolution

**Figure 2:**
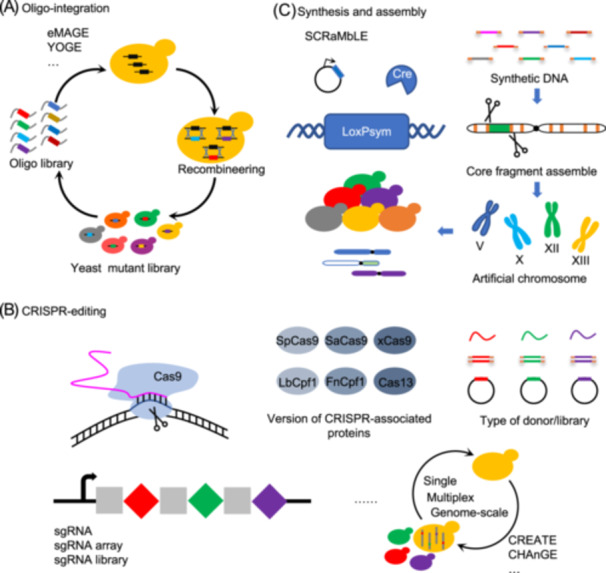
Template‐dependent synthetic evolution. (A) Oligo‐integration. Using oligonucleotide donors, eMAGE and YOGE are utilized to induce chromosome site‐specific alterations in yeast cells, enhancing the population of necessary mutations. (B) CRISPR‐editing. The CRISPR technology offers an unprecedented tool for yeast genome engineering. The development of CREATE and CHAnGE allows yeast to rapidly modify the genome and improve tolerance to growth inhibitors. (C) Synthesis and assembly. Chromosome rearrangements and alterations produced by the LoxP‐mediated evolution (SCRaMbLE) system are critical in the efficient design of sites that promote genotypic variation and speeded up genome evolution. CHAnGE, CRISPR–Cas9‐ and homology directed‐repair (HDR)‐assisted genome‐scale engineering; CREATE, CRISPR‐enabled trackable genome engineering; eMAGE, eukaryotic multiplex automated genome engineering; SCRaMbLE, synthetic chromosome rearrangement and modification by LoxP‐mediated evolution; YOGE, yeast oligo‐mediated genome engineering.

Biological systems have evolved two main mechanisms to repair genomic insult and damage: homology‐directed repair (HDR) and non‐homologous end joining (NHEJ). *S. cerevisiae* prefers to conduct templated precise repair via homologous recombination with an exogenous donor DNA molecule. Thus, template‐dependent synthetic evolution tools and strategies have been preferentially developed in recent decades. Templated donors used for synthetic evolution engineering mainly contain large double‐stranded DNA constructs and short single‐stranded oligonucleotides. Short oligonucleotides‐directed evolution engineering has been used more and more since oligonucleotides are inexpensive and easily obtained^34^. Chromosomal site‐specific alterations might be achieved in yeast using oligonucleotide donors, but the efficiency still has to be improved^35^, ^36^. Recent synthetic biology technologies have boosted the efficiency and diversity of genetic alteration^37^, allowing for more efficient and directed creation of diversity toward specific targets to expedite evolution (Figure 2A). Yeast oligo‐mediated genome engineering (YOGE) is a good illustration of how high gene modification rates can be accomplished with only a few hundred cells being screened^38^. Similarly, eukaryotic multiplex automated genome engineering (eMAGE) can easily diversify genomic DNA based on synthetic oligonucleotides in *S. cerevisiae* ^39^. Although these approaches are multiplexable, and subsequent cycles can enrich the population for the desired mutations, genome‐wide engineering has yet to be shown^38^, ^39^.

With breakthroughs in DNA synthesis and high‐throughput sequencing technology, various donor DNA libraries can be created and resynthesized according to arbitrary design principles, allowing for faster genome‐wide modification. Furthermore, the rapid advancement of genome editing technology, especially the CRISPR/Cas system, has provided unparalleled tools for yeast genome evolution^40^, ^41^, ^42^. CRISPR‐enabled trackable genome engineering (CREATE) was developed using a combination of CRISPR/Cas9 and massively parallel oligomer synthesis and has since been applied to site saturation mutagenesis for protein engineering, as well as adaptive evolution in bacteria, with preliminary results in *S. cerevisiae* ^43^, ^44^. Moreover, a precise and trackable edit engineering technique, named CHAnGE, was also created with the help of CRISPR/Cas9 and single‐nucleotide‐mediated HDR, allowing for the rapid evolution on a genome scale and improving tolerance to growth inhibitors of *S. cerevisiae* ^45^. Previous studies have also investigated and employed large‐size donors for robust directed evolution. With the assistance of error‐prone PCR and Cas9‐mediated genome editing, CasPER allowed the integration of large‐scale donor‐mediated variant mutant libraries into multiple genomic sites with efficiencies of almost 100.0%^46^. Moreover, a homology‐integrated CRISPR (HI‐CRISPR) strategy enabled the insertion of a 100‐bp dsDNA mutagenized homologous recombination donor into target locations for multiple gene knockouts and genome evolution^47^. Si et al. also developed an automated multiplex genome‐scale engineering in a completely automated manner using CRISPR‐Cas and a full‐length cDNA library^48^ (Figure 2B).

With the deepening knowledge of yeast genome information and the development of toolkits for large‐scale reading and writing of DNA, chromosomal rearrangements and artificial synthesis give a novel strategy for producing genotype varieties and accelerating genome evolution^49^. As previously reported, the design of artificially manufactured and fully customizable *S. cerevisiae* 2.0 (Sc2.0) genomes was accomplished, allowing experimentalists to direct genome evolution using a “bottom‐up” strategy^50^. In the Sc2.0 project, the synthetic chromosome rearrangement and modification by the LoxP‐mediated evolution (SCRaMbLE) system plays a critical role in the production of chromosomal rearrangements, generating diverse genomic alterations at designed loci at high efficiency^26^, ^27^, ^51^, ^52^. ReSCuES, a reporter system of SCRaMbLE, was developed, enabling easy identification and characterization of SCRaMbLEd cells^28^. Moreover, a light‐controlled SCRaMbLE system, named L‐SCRaMbLE, was further developed, which can tightly regulate recombinase activity by red light^53^. The SCRaMbLE system allows for rapid genome diversification, which opens up massive possibilities for strain evolution^54^, ^55^, ^56^ (Figure 2C).

### Template‐free synthetic evolution

**Figure 3:**
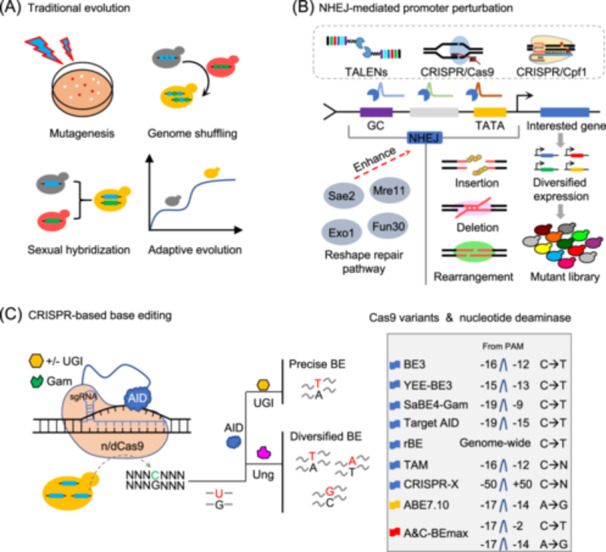
Template‐free synthetic evolution. (A) Traditional evolution. Mutagenesis, genome shuffling, sexual hybridization, and adaptive evolution are four common evolutionary techniques. (B) NHEJ‐mediated promoter perturbation. By introducing random mutations, insertions, deletions, and rearrangements at the target spot in the error‐prone NHEJ repair, the TALENs system, CRISPR/Cas9, and CRISPR/Cpf1 are used to execute directed evolution of the yeast genome, boosting genome variation and improving genome evolution. (C) CRISPR‐based base editing. Activation‐induced AID could effectively and permanently convert C•G to T•A base pairs in yeast by utilizing damaged Cas9. This method offers a powerful platform for mutagenesis and genomic evolution. CRISPR, Clustered Regularly Interspaced Short Palindromic Repeats; NHEJ, non‐homologous end joining; rBE, random base editing; TALENs, transcription activator‐like effector nucleases.

Template‐mediated genome engineering with the CRISPR/Cas system as well as other nuclease tools has greatly facilitated the development of synthetic evolution. However, template‐free synthetic evolution appears to be more convenient than donor‐mediated genome engineering^57^. For past decades, traditional evolution technologies (Figure 3A), such as mutagenesis, genome shuffling, sexual hybridization, or adaptive evolution, are template‐free; nevertheless, the efficiency and diversity of genetic alteration were always hard to be satisfied to obtain the desirable outcomes. Fortunately, the CRISPR/Cas system and many other nuclease tools were used to develop novel template‐free synthetic evolution strategies that do not require chemical synthesis to evolve chimeric donors in vitro. In the absence of exogenous DNA donors, *S. cerevisiae* preferentially employed the NEHJ pathway for repairing genome DNA double‐strand break (DSB) damages. Random mutagenesis was introduced into target locations among the error‐prone NHEJ repair, thus increasing genomic variants and enhancing genome evolution (Figure 3B)^58^. Zhang et al. reported the transcription activator‐like effector nucleases (TALENs)‐assisted multiplex editing (TAME) system, allowing for the directed evolution of yeast genome with iterative cycles of TAME in a short amount of time^59^. TAME provids a powerful platform for enhancing various stress tolerances of laboratory and industrial *S. cerevisiae*, while the efficiency of NEHJ‐based mutagenesis still needs to be improved^59^, ^60^. Modification of DSB repair proteins is an efficient approach for increasing the efficiency of NHEJ‐based mutagenesis. Wang et al. devised a novel mutagenic genome editing (mGE) technology using an error‐prone DNA repair protein and genome editing tools^58^. The mGE toolkit allows for the diversification of gene expression and enhancement of genome evolution by introducing varied mutagenesis at the targeted promoter.

Although nuclease‐induced DSBs are convenient for synthetic evolution, they also result in extra genomic damage and cell death^58^, ^61^. The invention of Cas9 nickase (nCas9) and cytidine deaminase‐assisted base editing technology has recently allowed for highly effective single‐nucleotide conversion and genomic disruption without DSB or donor DNA (Figure 3C)^62^. With the help of an impaired Cas9, the activation‐induced cytidine deaminase (AID) allows for efficient and persistent C•G to T•A base pair conversion in yeast^63^. Liu et al. reported a target‐AID base editor system enabling C‐to‐T substitutions and employed it to generate in situ nucleotide changes, thereby diversifying stress tolerances in *S. cerevisiae* ^64^. To expand the genome‐targeting scope and site selectivity of base editing, massive Cas9 variants that recognize diverse protospacer adjacent motifs (PAMs) were generated and used for developing a series of high‐precision base editing tools^65^, ^66^. In addition to the defective Cas9 nuclease, various targeted proteins, such as eukaryotic helicase, T7 RNA polymerase, and others, were also used to construct base editing tools for *S. cerevisiae* ^67^, ^68^. With the aid of an error‐prone T7 RNA polymerase, a directed evolution (CRAIDE) system was established in *S. cerevisiae* with dramatically enhanced mutation efficiency without the usage of in vitro provided and pre‐programmed repair donors^69^. Huang et al. developed MutaT7^trans^, a targeted mutagenesis method that dramatically increases the rate of mutation and produces mutations in yeast across all four nucleotides. The percentage of non‐C‐to‐T mutations using this mutagenesis method was 10.0–11.0 times higher than that of evolutionary tools based on cytidine deaminase^70^. Except for targeted proteins, the optimized cytidine deaminases have also been used a lot^66^. The APOBEC‐nCas9‐Ung system, in particular, can generate C‐to‐A transversion in *E. coli* and C‐to‐G transversion in mammalian cells, considerably expanding the range of base editing applications^71^. Then, by replacing the human Ung in glycosylase base editor (GBE) with Ung1 from *S. cerevisiae*, the APOBEC‐nCas9‐Ung1 system was recreated with improved C‐to‐G base editing efficiency and purity^72^. In addition, the error‐prone DNA polymerase was used to create the flexible EvolvR system, which provides a platform for performing directed evolution in *S. cerevisiae* for engineering applications^73^. Moreover, recent investigations in *S. cerevisiae* have described multiple CRISPR nickase‐assisted base editing techniques, allowing *S. cerevisiae* cells to be precisely edited at genome‐wide levels in a matter of days^74^, ^75^. Pan et al. devised a random base editing (rBE) system using the fusion of ssDNA‐binding protein and cytidine deaminase. The rBE allows for genome‐wide C‐to‐T mutations and accelerates *S. cerevisiae* genome development^76^. Recently, Chen et al. reported a programmable platform, known as helicase‐assisted continuous editing (HACE), which could introduce long‐range and locus‐specific hypermutation in mammalian cells^77^. This strategy may also be applicable for accelerating genome evolution in *S. cerevisiae*. By synthetically optimizing the retrotransposon Ty1, Nathan Crook et al. also developed an in vivo continuous evolution (ICE) strategy, which can generate up to 1.6 × 10^7^ l^−1^ of mutant libraries per round. When combined with growth selection, this method allows the evolution of single enzymes, multi‐gene pathways, and global transcriptional regulators^25^. OrthoRep, an orthogonal DNA polymerase‐plasmid pair in yeast, was described by Arjun Ravikumar et al. It stably mutates approximately 100,000 times faster than the host genome in vivo, above the genomic replication error threshold that results in single‐generation extinction^33^. Overall, the template‐free synthetic evolution provides a powerful platform for mutagenesis and genome evolution.

### Transcriptional regulation‐based synthetic evolution

Both template‐dependent genome engineering and template‐free genomic mutagenesis are reliable and highly generalizable methods for accelerating genome synthetic evolution. However, these approaches frequently have to introduce genomic alterations. Furthermore, reshaping metabolic pathways and accelerating genome evolution demands tuning gene expression levels to link phenotypes—from deletion to extreme overexpression^78^. In particular, transcriptional regulation allows massive‐scale gene regulation, exact timing, and position without genome changes^79^. Strategies of gene activation and interference without causing genetic changes mainly contained three aspects, including (1) RNA interference (RNAi), (2) synthetic transcriptional factors, promoters, and terminators, and (3) gene regulation mediated by customized nucleases, particularly dCas proteins.

**Figure 4:**
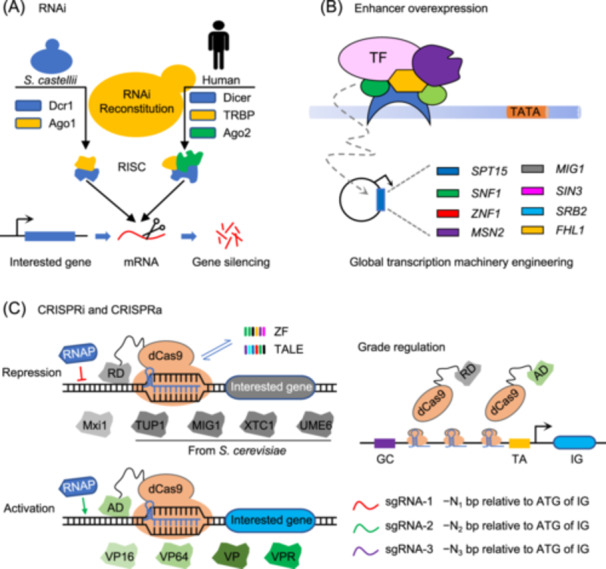
Transcriptional regulation‐based synthetic evolution. (A) RNAi. The heterologous RNAi pathway of human or *S. castelli* is introduced to reconstruct the RNAi mechanism to achieve the purpose of gene silencing in *S. cerevisiae*. (B) Enhancer overexpression. Transcriptional enhancers specify the precise time, level, and location of gene expression; global transcription machinery engineering (gTME) is an approach for reprogramming gene transcription to elicit cellular phenotypes. (C) CRISPRi and CRISPRa. The upregulation and downregulation of gene expression levels are mediated by customized nucleases, particularly dCas proteins. The efficacy of inhibition or activation is further increased by fusing numerous repressing or activating domains with dCas9. AD, activation domain; CRISPR, clustered regularly interspaced short palindromic repeats; CRISPRa, CRISPR activation; CRISPRi, CRISPR interference; dCas9, dead Cas9; IG, interested gene; RD, repression domain; RISC, RNA‐induced silencing complex; RNAP, RNA polymerase; TALE, transcription activator‐like effector; TF, transcription factor; TRBP, Trans‐activation response RNA‐binding protein; VPR, VP64‐P65‐RTA; ZF, zinc‐finger.

Although RNAi is a conserved regulatory system in eukaryotes, it is absent in *S. cerevisiae*, presumably because it cannot coexist with a useful dsRNA “killing virus”^80^. Thus, except for *S. cerevisiae*, nearly all eukaryotic species could employ the powerful RNAi tool for strain engineering^81^, ^82^. This convenient tool for sequence‐specific gene suppression in *S. cerevisiae* is made possible by the recent reconstruction of the RNAi machinery by introducing heterologous RNAi pathways from humans or *S. castelli* (*Naumovozyma castelli*)^83^, ^84^, ^85^. RNAi has been used more frequently in yeast engineering with the aid of RNAi reagent cassettes and genome‐scale library design, providing unique benefits like dynamic regulation, multigene optimization, as well as genome‐scale engineering and evolution^81^. The effects of RNAi could be nonspecific and ineffective, although it is a versatile tool for understanding gene function and reshaping metabolic pathways. Moreover, RNAi prefers to induce gene silencing rather than gene activation, which is necessary for enhancing cell phenotypes and accelerating strain evolution^79^, ^86^ (Figure 4A).

With the development of programmable nucleases like zinc‐finger (ZF), TALEs, and nuclease‐dead Cas9 (dCas9), global controlling the expression of the target gene is made very simple (Figure 4B). These gene regulatory technologies were based on the ability of programmable nucleases to target and attach to specific DNA sequences and overexpression of transcription enhancers. Artificial transcription factors (TFs) combining three ZF DNA‐binding domains with a transcriptional effector domain enabled recognition of 9‐bp of DNA sequences and semi‐rational activation or repression of a gene. When ZF domains reach up to six, artificial TFs might specifically recognize 18‐bp targeted genomic regions and result in rational gene disruptions. Similarly, the programmed TALE proteins can identify and bind particular and nonspecific DNA sequences by adjusting the number of TALE binding sites. Synthetic TFs allow for the natural activation or artificial suppression of gene expression through fusion with transcriptional domains. By fusing more than 100,000 ZF proteins with either a transcription activation or repression domain, Park et al. constructed randomized libraries of artificial TFs and generated various phenotypic changes^20^. Although TFs mediated by ZF and TALEs could create precise and non‐mutagenesis expression regulation, the arduous design and extensive costs required to establish new ZFs and TALEs severely constrain their applications.

DNA targeting has become more adaptable thanks to recent advancements in the programmable CRISPR/Cas9 system. Nuclease‐dCas9 lost its ability to cut DNA but retained its DNA‐binding capability^87^. As a result, it may sterically prevent the RNA polymerase from binding to elongating in the area surrounding the promoter of interested genes, then resulting in transcription repression. Based on dCas9, the straightforward but effective CRISPR interference (CRISPRi) method has been successfully created to reduce the expression level of the interested genes (Figure 4C). In addition, the fusion of a repressor domain, such as Mxi1, with dCas9 protein can further strengthen the capacity of transcriptional repression. Compared with human Mxi1, repression domains of transcriptional regulatory proteins from *S. cerevisiae* like TUP1, MIG1, XTC1, and UME6 enabled a further strengthened level of down‐regulation^88^. On the other hand, the combination of transcriptional activators like VP16, VP64, VP, and VPR with dCas9 could enhance the recruitment of RNA polymerases and cause gene expression up‐regulation (Figure 4C). Moreover, multiple repression or activation domains fused to dCas9 further improved the inhibition or activation efficiency. Traditional CRISPRi and CRISPRa have been limited to binary on/off regulation, which ignores instructional and potentially useful intermediate levels of gene expression. In a recent study, Deaner et al. designed flexible sgRNA target sites throughout a yeast promoter region to generate graded gene expression with a wide dynamic range^22^. To optimize metabolic pathways and obtain the desired phenotype, it always requires multiplex, even genome‐wide transcriptional regulation engineering. According to empirical design principles, McGlincy et al. developed a thorough yeast CRISPRi library, produced genome‐scale CRISPR interference, and obtained thorough phenotypic profiling^89^. Increasing the version of dCas9 proteins, such as dSpCas9, dSaCas9, dSt1Cas9, and dLbCpf1, is an alternative method to generate multiplex transcriptional regulation^88^.

The exact time, degree, and location of gene expression are all specified by transcriptional enhancers. Because enhancers contain activator and repressor binding sites for many proteins that each exerts subtle, context‐dependent regulation of enhancer activity, it has been difficult to disentangle and characterize the components of enhancer activity in multicellular eukaryotic development. Recent advancements in synthetic biology have given researchers the previously unheard‐of capacity to explore gene regulation by enabling nearly limitless creation and modification of regulatory components and networks. Here, we highlight recent studies that show how useful synthetic biology is for examining enhancer activity during development and evolution. These investigations unequivocally demonstrate that synthetic biology offers a means of reversing and reengineering transcriptional control in animal genomes, which holds great promise for comprehending evolution^79^.

The technique known as global transcription machinery engineering (gTME) aims to rewire gene transcription to produce biological characteristics that are crucial for technological uses. Dominant mutations resulting from selection and mutagenesis of the transcription factor Spt15p provided the required phenotypes. The three distinct mutations in the *SPT15* gene, serine replacing phenylalanine (Phe177Ser), Tyr195His, and Lys218Arg, combined to produce the desired phenotype. Therefore, gTME can offer a path to intricate phenotypes that are difficult to obtain by conventional means^21^.

Snf1 protein kinase, a member of the SNF1/AMPK family, controls the transcription of a wide range of genes, alters the activity of metabolic enzymes, and governs several cellular developmental processes. In addition, the Snf1 protein kinase plays a crucial role in controlling how yeast reacts to stress. *SNF1* overexpression improved the cell tolerance and fermentation ability of baker's yeast in freezing conditions. To provide cells resistance to freezing, *SNF1* overexpression changed the levels of leucin, serine, proline, isoleucine, homocitrulline, arginine, glycerol, palmitic acid, lysophosphatidylethanolamine (LysoPE), and lysophosphatidylcholine (LysoPC) prior to freezing. With elevated LysoPC and LysoPE content, *SNF1* overexpression maintained a relatively high level of proline, lysine, and glycerol after freezing^90^.

## APPLICATIONS OF SYNTHETIC EVOLUTION

**Figure 5:**
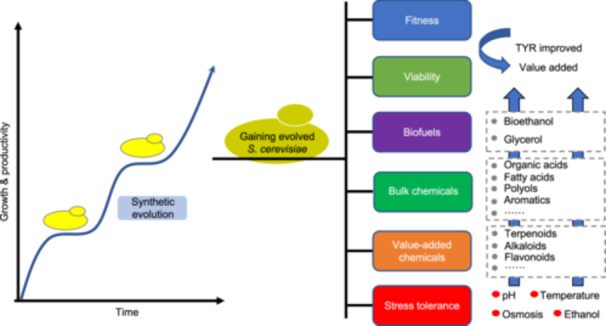
Application of synthetic evolution in *S. cerevisiae*. To meet commercial demands, industrial yeast often needs to be engineered to gain multiple stress tolerance and achieve specific metrics, such as titer, yield, and rate (TYR). The advancements in synthetic evolution have significantly increased the ability to rapidly create novel yeast with enhanced robustness and optimized fermentation performance. On the one hand, synthetic evolution has been proven as a powerful strategy for increasing the fitness and viability of industrial yeast and enhancing various fermentation stress tolerance, including high osmosis, ethanol, low pH, and extremely high or low temperatures. Improving stress tolerance and robustness allows for more efficient production and ultimately makes a process more economically viable. On the other hand, synthetic evolution is also a potent tactic to improve *S. cerevisiae*'s metabolic performance in the synthesis of bulk chemicals, value‐added compounds, and biofuels. These range from low‐value, high‐volume products like ethanol and other biofuels to high‐value, low‐volume items like natural triterpenoid compounds.

Multiple genes interact with environmental conditions to determine the phenotypic of biological systems. Typically, synthetic evolution technologies aim to enhance and refine the characteristics and uses of biological systems, like enhancing resistance to environmental changes and enhancing product yield. As a result of recent advancements in the construction of increasingly intricate novel functions at the level of metabolic pathways, population diversity and adaptation to upcoming problems have increased in *S. cerevisiae* for improving the performance of biomanufacturing^18^ (Figure 5 and Table 2).

**Table 2:** Applications of synthetic evolution strategies in *S. cerevisiae*.

Phenotype	Strategy	Result	Ref.
***Enhancing stress tolerance***			
Alkali and salt	SCRaMbLE	Increasing alkali tolerance with pH8.0 and tolerance of 0.4 M NaCl	^54^, ^91^
Acetic acid and furfural	CHAnGE	Improving acetic acid tolerance 20.0‐fold; growth increased by 8.1‐fold in 10.0 mM furfural	^45^
High temperature and osmosis	TAME	1.2‐fold to 1.3‐fold increases in fermentation capacities at 400.0 g/l glucose and/or 42°C	^60^
Lactic acid	Metabolic engineering and adaptive evolution	Increasing l‐lactic acid tolerance with 60.0 g/l	^92^
High temperature and ethanol	Target‐AID base editing	More than 2.5‐fold and 3.0‐fold increases in fermentation parameters under thermal and ethanol stress, respectively	^64^
***Improving metabolic performance in the production of biofuels and bulk chemicals***			
Ethanol	mGE	Ethanol production increased by about 9.5% compared to the wild type	^58^
Lycopene/n‐butanol	POTAC	The production of lycopene and n‐butanol in the host strain INVSc1 enhanced by 10.0 and 100.0 times	^93^
Ethyl acetate	Multiple CRISPRi	The yield of ethyl acetate increased by 3.8 times	^94^
Glycerol/3‐dehydroshikimate	STEPS	The glycerol titer increased by 5.7 times; the titer of 3‐dehydroshikimate increases 7.8 times	^22^
Isoprenoid	Error‐prone PCR and Cas9‐mediated genome integration	Resulting in an 11.0‐fold increase in isoprenoid production	^46^
***Improving metabolic performance in the production of value‐added chemicals***			
β‐carotene	eMAGE	The synthesis of β‐carotene increased by nearly 7.0 times	^39^
CAPE/CAPA	Combination of rational engineering and SCRaMbLE	The yield of CAPE and CAPA reaching 417.0 μg/l and 1081.0 μg/l, respectively	^95^
Lycopene	Combination rDNA arrays, SCRaMbLE, and optimization of culturing conditions	Lycopene yield increased by 129.5‐fold	^96^
PPD	CRISPRi‐based customized metabolic flux system	PPD production increased by 14.4‐fold in shake‐flask fermentation and 5.7‐fold in a long‐term batch‐fed fermentation	^97^
Taxadiene	Pathway balance	Resulting in taxadiene production in *S. cerevisiae* of 528.0 mg/l	^98^

CAPE, caffeic acid‐derived phenethyl ester; CAPA, caffeic acid‐derived phenethyl amide; CHAnGE, CRISPR–Cas9‐ and homology directed‐repair (HDR)‐assisted genome‐scale engineering; eMAGE, eukaryotic multiplex automated genome engineering; mGE, mutagenic genome editing; POTAC, pathway optimization by tuning antibiotic concentrations; PPD, protopanaxadiol; SCRaMbLE, synthetic chromosome rearrangement and modification by LoxP‐mediated evolution; STEPS, Systematically Test Enzyme Perturbation Sensitivities; TAME, TALENs‐assisted multiplex editing.

### Enhancing environmental stress tolerance

*S. cerevisiae* has been widely employed in complex environmental conditions as a model for fundamental biological studies and a vital cell factory^99^. Developing evolutionary biology that exploits advantageous mutations to make the strain more adaptive to the complex environmental conditions^100^. SCRaMbLE system has been introduced in the synthetic yeast genome, which enables the generation of a yeast library with massive structural variations and drives phenotypic evolution. Through SCRaMbLE, multiple stress tolerance has been enhanced, such as alkali (pH 8.0), salt stress, and 5‐fluorocytosine^54^, ^91^. Furthermore, the use of natural genetic elements or the introduction of heterologous pathways to perform directed evolution, such as the development of CRISPR/Cas9 systems, is frequently used for efficient knock‐in and knock‐out in a marker‐free manner to perform multiple gene modifications quickly and efficiently. *S. cerevisiae* is engineered to be more resistant to environmental stress, hence improving strain performance^101^. CHAnGE approach that employs CRISPR/Cas9 and HDR increased the resistance of *S. cerevisiae* cells to furfural and possessed single nucleotide accuracy^45^. Overexpressing *OLE1* in a CRISPR‐Cas‐based *S. cerevisiae* gene activation library after 5 passages at 42°C improved yeast heat resistance^102^.

The proofreading faulty DNA polymerase delta variation can boost the mutation rate in the lag chain synthesis process, introducing non‐lethal mutations with growth advantages in various stress settings into the genome. The genome rearrangement approach improved *S. cerevisiae*'s environmental stress tolerance^103^. Mitsui et al. employed CRISPR/Cas9 technology to break the δ‐sequence, resulting in large‐scale rearrangements, including gene amplification, translocation, and deletion. Under stressful conditions, the genome evolved toward a more favorable phenotype. The DNA‐repaired δT8‐292 strain thrived well at 39°C and maintained high cell viability under acidic and high ethanol conditions, even after heat stress^104^. Three rounds of protoplast fusion were carried out, with the ethanol content of the culture dish and the incubation temperature for selection gradually increasing in each round. After three rounds of genomic screening, the top‐performing strain, F34, was identified. It could totally utilize 20.0% (w/v) glucose at 45–48°C, create 10.0% (w/v) ethanol, and withstand 25.0% (v/v) ethanol stress^105^. Cheng et al. employed CRISPR/Cas9 to insert the loxPsym sequence into numerous genomic locations simultaneously. The loxPsym site in SparLox83R significantly improved the SCRaMbLE system via the ReSCuES system, resulting in a variety of large‐scale genome rearrangements dominated by interchromosomal events. Translocations and recurrent events have been observed to improve noconazole tolerance. In the Sc2.0 project, the haploid strain (JDY541) harboring synIII was crossed, resulting in rapid adaptation to acetic acid tolerance^106^. Fleiss et al. reported a novel technology to distinguish between the phenotypic effects of massive chromosomal rearrangements and point mutations. They present a new use of the CRISPR/Cas9 technology that enables the highly effective generation of multiple concurrent reciprocal translocations in the yeast genome as well as specifically targeted translocations. The addition of four tandem repeats of a 76 bp segment in the promoter region of *SSU1* originating from the *ECM34* locus in the translocated lab strain (YAF158) resulted in increased sulfite resistance with a minimal inhibitory concentration of 7.0 mM^107^.

Genome‐wide transformation transferred the target trait genetic components from the donor strain to the recipient strain, improving the heat tolerance of the *S. cerevisiae* KEA24 strain in earlier studies^108^. Vanmarcke et al. discovered that a new *AST2* mutation improved the MD4 strain's tolerance to 5‐hydroxymethylfurfural (HMF) and acetic acid. High acetic acid levels caused a rise in acetaldehyde levels, which were then transformed into ethanol by *AST2* ^109^. Gonçalves et al. identified the genetic traits of brewer's yeast domestication by analyzing brewing‐related genes and doing genome‐wide scanning. The *RTM1* gene, which was used to characterize the domesticated strain, was found to be located at the sucrose consumption site and was resistant to the inhibitory chemicals found in molasses^110^. Sezmis et al. employed DBY15084 as a host strain to generate mutants by adaptive evolution. The *TOH1‐*deficient mutant promoted growth and fermentation in high‐glucose environments, whereas the *KRE6* mutant improved adaptation in low‐glucose environments^100^. Based on genome sequence analysis, Firdaus‐Raih et al. discovered that genes encoding cold tolerance‐related proteins, like antifreeze proteins (AFPs), constructed a cold adaptation system in which AFPs were expressed at low temperatures in response to persistently cold settings ^111^.

### Improving metabolic performance in the production of biofuels and bulk chemicals

The demand for renewable fuels and chemicals has increased because of worries about global warming and unsustainable consumption^112^, ^113^. Lignocellulosic biomass has the ability to produce fuel ethanol and chemicals^114^. *S. cerevisiae* is a versatile cell factory that converts renewable biomass into biofuels and biobased compounds. However, during fermentation, lignocellulosic hydrolysates, such as furfural, phenols, and carboxylic acids, have a stronger inhibitory effect on the development and fermentation of industrial *S. cerevisiae* ^115^. Furfural is one of the primary inhibitors, causing the formation of reactive oxygen species, reducing the function of critical enzymes in several metabolic pathways, and eventually leading to programmed cell death^116^. Reverse genetics was used to uncover genes and transcriptional regulators that may be involved in tolerance inhibitors. These genes and transcriptional regulators react to metabolic pathways and alter upregulation and downregulation to increase tolerance^117^. The two are linked using reverse metabolic engineering, which is based on mutagenesis and evolution concepts, to increase *S. cerevisiae*'s ethanol production capability^118^.

The CRISPR system has great potential to facilitate the occurrence of high‐efficiency multiple gene knock‐ins and knock‐outs without the need for a selection marker. Thus, the CRISPR system has been used very often for conducting metabolic engineering processes, such as the introduction of multiple gene pathways^119^ or suppression of multiple gene expression to reduce by‐product formation^120^, ^121^. Multi‐expression based on gRNA and simultaneous expression of multiple homologous Cas9 or dCas9 proteins can achieve multiplex orthogonal editing or multifunctional combinatorial metabolic engineering of CRISPR systems^122^. Lian et al. reported the pathway optimization by tuning antibiotic concentrations (POTAC) strategy, which is used for multiple genome integration, attaches to plasmids with specific markers, and modulates plasmid copy number based on antibiotic concentration. By employing POTAC, the production of lycopene and n‐butanol in the host INVSc1 was enhanced by 10.0 and 100.0 times, respectively^93^. Lignocellulosic biomass, such as xylose, can provide renewable raw materials for biofuel production^114^. Shi et al. used the delta integration CRISPR‐Cas (Di‐CRISPR) system to induce DSBs at the delta (δ) position of the endogenous sequence Ty (yeast transposon) element for multiple copies of DNA. Compared to the single‐copy integrated strain, 18 copies of the 24 kb DNA fragment carrying the xylose utilization and 2‐3‐butanediol (BDO) synthesis pathway were integrated, increasing the engineered strain CEN.PK2‐1C's xylose consumption and BDO production to 12.5 g/l^123^. Industrial yeast strains’ chromosomes typically contain numerous copies. The creation of an efficient CRISPR/Cas9 system for multi‐genome alteration projects is a valuable tool for the development of cell factories^124^, ^125^. Lian et al. used the optimized CRISPR/Cas9 system to perform metabolic engineering, knocking out four alleles with a 100.0% efficiency in the diploid strain Ethanol Red and the triploid strain ATCC 4124, and found an increase in xylose utilization (about 0.2 and 0.4 times) and lactic acid production (about 2.3 and 1.2 times)^126^.

Since Jiang et al. proved that the native CRISPR/Cas9 system of *Streptococcus pneumoniae* has multiple application potential in 2013, a number of multiplex application studies of the CRISPR system in *S. cerevisiae* based on gRNA multiexpression strategy were developed. Thus, *S. cerevisiae* chassis cells can be modified, ultimately increasing the yield of the target product or changing the characteristics of the cell^127^. Zalatan et al. integrated the aptamer and recognition area using a multi‐SGRNA expression cassette method and sgRNA engineering. Following the fusion expression of the ligand and transcription factor, the three strongest target genes were activated or repressed simultaneously. The bacterial violet biosynthetic pathway was heterologously expressed in yeast and then metabolized in *S. cerevisiae* strain yML025 to yield four distinct violet compounds^128^, ^129^. CRISPR/Cas9 targeted gene editing was used to manipulate traits to enhance the metabolic ability of the strain. Löbs et al. used multiple CRISPRi regulation in the central carbon pathway and knocked out *ACO2b*, *SDH2*, *RIP1*, and *MSS51* genes at the same time. Compared with previous studies, the yield of ethyl acetate was increased by 3.8 times^94^. Deaner and Alper identified the rate‐limiting stage in the metabolic route by controlling and grading gene expression pathway enzymes with dCas9. *GPD1/GPP1* was overexpressed in the GDTY strain, and the titer rose by 5.7 times following two rounds of iterative optimization. Finally, the glycerol titer was enhanced from 4.89 to 28.0 g/l. Overexpression of *TKL1* enhanced the titer of 3‐dehydroshikimate by 7.8 times, reaching 126.4 mg/l^22^. Jakočiūnas et al. used CRISPR/Cas9 to introduce DSB at genomic targets and native HDR to integrate mutagenized 300‐600 bp linear DNA donors. They also used the method to direct the evolution of two key metabolic enzymes (ERG12 and ERG20) in the mevalonate pathway of *S. cerevisiae*, resulting in an 11‐fold increase in isoprenoid production^46^.

### Improving metabolic performance in the production of value‐added chemicals

To create various variations in *S. cerevisiae*, Barbieri et al. created and used eMAGE to diversify a heterologous β‐carotene pathway. When nucleosome disfavoring poly (dT) 20 sequences were introduced to the promoters for *crtE* and *crtI*, the synthesis of β‐carotene increased by nearly seven times^39^. SCRaMbLE effectively enhances *S. cerevisiae*'s fermentation capabilities. Regarding the β‐carotene route, the SCRaMbLE‐in strain LWy252 had a total β‐carotene output of approximately 500.0 μg/l, which is twice that of the HO‐integrated strain LWy212^29^. After combining the rational route engineering approach with the library‐based SCRaMbLE method, the evolved yeast generated 417.0 μg/l of Caffeic acid‐derived phenethyl ester (CAPE) and 1081.0 μg/l of Caffeic acid‐derived phenethyl amide (CAPA), respectively^95^. After integrating the rDNA arrays, inducing SCRaMbLE, and optimizing the culturing conditions, the final yeast strain, YSy222, demonstrated a 129.5‐fold increase in lycopene yield when compared to its parental strain^96^. Moreover, using the SCRaMbLE system integrated into the Sc2.0 synthetic yeast, Zhang et al. created a simple evolution platform to screen strains with increased geraniol yield and verify the causal role of pertinent genetic targets. The end strain produced more than 30 times as much 8‐hydroxygeraniol (G8H) as the first strain thanks to the combination of the designed chassis, enhanced biosynthetic pathway, and use of G8H^130^.

Plant terpenoids are the most abundant secondary metabolites found in natural products. Their structure and functions are different. Sterols, carotenoids, and other hormones have a tight relationship to human nutrition and health. They are frequently utilized as raw ingredients in medicine, food, and the chemical sector for the production of anticancer medications, cosmetics, chemicals, and biofuels^131^, ^132^. Constructing terpenoid microbial cell factories to boost the production of high‐value‐added products is an important step toward achieving sustainable development^133^. The orthogonal three‐function (knock‐in, knock‐down, and knock‐out) CRISPR/Cas9 system can be employed for multiple genome editing, in contrast to ZNFs and TALENs. For instance, it has become one of the most potent methods for combinatorial metabolic engineering to enhance terpenoid production by genome editing in microbial hosts^134^, ^135^. In comparison to *E. coli*, *S. cerevisiae* can synthesize acetyl‐CoA and NADPH, which are essential precursors in the production of terpenoids. The CRISPR/Cas9 system has matured in *S. cerevisiae* genome editing, and it has emerged as the main host strain for terpene production^136^.

Protopanaxadiol (PPD) is an essential precursor for the production of ginseng/notoginsenoside, which has numerous anti‐cancer, anti‐inflammatory, and antioxidant properties. PPD is extracted and purified from ginseng, but the content is rare, making it difficult to meet market demand. Using *S. cerevisiae* as the starting strain, Lim et al. employed CRISPRi to control the metabolic flux of the yeast and dCas9 to target sgRNA (sgRNA1‐5) that inhibited the expression of *ERG7*. In *S. cerevisiae*, the conversion of 2,3‐oxidosqualene to DD‐II and PPD was accelerated. With CEN.PK2‐1D as the control strain, the PPD titer of the PPD‐A strain grew by 14.4 times after 48 h of shaking flask fermentation, while the PPD titer of dCas9‐sgRNA‐mediated *ERG7* inhibition increased by 5.7 times after 216 h of batch fermentation^97^. Zhu et al. constructed a PPD‐producing strain by modularly integrating *S. cerevisiae* BY4742's multi‐copy sites. CRISPR/Cas9 gene editing technology was used to regulate yeast cells’ metabolic network, up‐regulate the key endoplasmic reticulum factor *INO2* gene to promote endoplasmic reticulum proliferation, down‐regulate *ERG7* and *LPP1* to inhibit the competitive, and overexpression of sterol transcription regulator *UPC2.1* to maintain cell homeostasis. The PPD titers of strain BY‐V in the shaking flask and 5.0 l bioreactor were 1.6 and 15.9 g/l, respectively, the highest reported value at present^137^.

Triterpenoids are the most abundant type of natural products. Betulinic acid, oleanolic acid (OA), and ursolic acid (UA) have significant therapeutic properties. They are most commonly found in plant leaves, flowers, and fruits, but their extraction efficiency is exceedingly low^138^, ^139^, ^140^. Previous studies have shown that *S. cerevisiae* is easier to conduct than *Yarrowia lipolytica*, with UA and OA titers of 123.3 and 155.6 mg/l in a 5 l fermenter, respectively^141^, ^142^, ^143^. The MVA pathway of *S. cerevisiae* contains the majority of isopentenyl pyrophosphate and dimethylallyl pyrophosphate, which are essential for terpenoid synthesis^144^. Jin et al. expressed and optimized CrAS, CrAO, and AtCPR1 using the C30 platform strain, resulting in UA and OA titers of 7.4 and 3.0 mg/l, respectively. By optimizing the copy number of *ERG1* and *CrAS* and down‐regulating *ERG7* expression, UA and OA titers were boosted by 50.0 times, reaching 483.4 and 163.8 mg/l, respectively. The UA and OA titers were grown to 1132.9 and 433.9 mg/l by fed‐batch fermentation of yeast strain UO19 in a 3.0 l fermentor, respectively, resulting in the highest UA titer reported until now^145^. With an increasing number of cancer cases, the development of effective and low‐cost anti‐cancer medications is critical. Taxol is a diterpene chemical that is commonly used to treat cancer. Reider Apel et al. developed a CRISPR/Cas9‐based cloning‐free toolkit to achieve chromosomal integration locus and promoter selection, thus accelerating genome evolution. By optimizing the expression of taxadiene synthase (TXS), a difficult but crucial enzyme for industry, they proved the usefulness of this toolbox and produced a 25.0‐fold increase in taxadiene production^146^. Karaca et al. recently used combinatorial design to find the best combination for producing taxadiene, an early‐step precursor to taxol. Combining the background strains SCIGS22a with 16 different episomal plasmids, the taxadiene titer in *S. cerevisiae* in shake flask culture peaked at 528.5 mg/l^98^.

## CONCLUDING REMARKS

With the advancements of exogenous gene expression and metabolic engineering, synthetic biology technologies like CRISPR/Cas9 have evolved in microorganisms to become the most efficient gene editing methods. Synthetic evolution aims at the progressive mutagenesis/selection of certain DNAs to efficiently produce goods on an industrial scale. The construction of microbial cell factories, particularly using *S. cerevisiae* as a chassis cell, has the advantage of a fast production cycle, ease of maintenance and manipulation, and suitability for large‐scale fermentation to produce chemicals, fuels, or pharmaceuticals in the bioengineering process. The CRISPR/Cas9 system's current molecular “toolbox” enables label‐free genetic manipulation and a number of quick and effective genetic modifications. These capabilities enable the system to adapt to environmental stresses like high pressure and temperature, ethanol, and high sugar levels in industrial fermentation processes. Base knockouts, insertions, substitutions, control over the expression of heterologous genes, precursor synthesis, and other changes to the carbon flow process all will contribute to the process's improvement. The CRISPR/Cas9 technique facilitates and speeds up the development of metabolic engineering by enabling gene editing and drastically cutting the time required to genetically modify cell factory constructs. Cas9 toxicity and off‐target effects are the main drawbacks and challenges of CRISPR/Cas technology. Efforts are being made to reduce off‐target effects by sequencing the entire genomes of microbial hosts. The excellent efficiency and specificity of sgRNA might also lessen mismatches. Cas9 toxicity can also be decreased by controlling the amount of intracellular Cas9 that is reached through temporary expression and a weak promoter. The ongoing advancement of novel CRISPR‐based synthetic evolution technologies enhances more conventional evolution approaches to hasten the creation of strains and maximize metabolite synthesis.
